# The prognostic nutritional index on postoperative day one is associated with one-year mortality after burn surgery in elderly patients

**DOI:** 10.1093/burnst/tkaa043

**Published:** 2021-03-01

**Authors:** Young Joo Seo, Yu-Gyeong Kong, Jihion Yu, Ji Hyun Park, Su-Jin Kim, Hee Yeong Kim, Young-Kug Kim

**Affiliations:** 1 Department of Anesthesiology and Pain Medicine, Hangang Sacred Heart Hospital, Hallym University College of Medicine, 12, Beodeunaru-ro 7-gil, Yeongdeungpo-gu, Seoul 07247, Republic of Korea; 2 Department of Anesthesiology and Pain Medicine, Asan Medical Center, University of Ulsan College of Medicine, 88, Olympic-ro 43-gil, Songpa-gu, Seoul 05505, Republic of Korea; 3 Department of Anesthesiology and Pain Medicine, National Medical Center, 245, Euljiro, Jung-gu, Seoul 04564, Republic of Korea

**Keywords:** Burn, Elderly patients, Prognostic nutritional index, Mortality, Nutrition, Geriatric patients

## Abstract

**Background:**

Burn injury in elderly patients can result in poor outcomes. Prognostic nutritional index (PNI) can predict the perioperative nutritional status and postoperative outcomes. We aim to evaluate the risk factors, including PNI, for one-year mortality after burn surgery in elderly patients.

**Methods:**

Burn patients aged ≥65 years were retrospectively included. PNI was calculated using the following equation: 10 × serum albumin level (g/dL) + 0.005 × total lymphocyte count (per mm^3^). Cox regression, receiver operating characteristic curve and Kaplan–Meier survival analyses were performed to evaluate the risk factors for postoperative one-year mortality.

**Results:**

Postoperative one-year mortality occurred in 71 (37.6%) of the 189 elderly burn patients. Risk factors for one-year mortality were PNI on postoperative day one (hazard ratio (HR) = 0.872; 95% CI = 0.812–0.936; *p* < 0.001), Sequential Organ Failure Assessment score (HR = 1.112; 95% CI = 1.005–1.230; *p* = 0.040), American Society of Anesthesiologists physical status (HR = 2.064; 95% CI = 1.211–3.517; *p* = 0.008), total body surface area burned (HR = 1.017; 95% CI = 1.003–1.032; *p* = 0.015) and preoperative serum creatinine level (HR = 1.386; 95% CI = 1.058–1.816; *p* = 0.018). The area under the curve of PNI for predicting one-year mortality after burn surgery was 0.774 (optimal cut-off value = 25.5). Patients with PNI ≤25.5 had a significantly lower one-year survival rate than those with PNI >25.5 (32.1% *vs* 75.9%, *p* < 0.001).

**Conclusions:**

PNI on postoperative day one was associated with postoperative one-year mortality in elderly burn patients. The postoperative one-year survival rate was lower in patients with PNI ≤25.5 than in those with PNI >25.5. These findings indicate the importance of identifying elderly burn patients with low PNI, thereby reducing the mortality after burn surgery.

Highlights

- This is the first study to assess the risk factors, including prognostic nutritional index, for one-year mortality after burn surgery in elderly patients.
- Prognostic nutritional index on postoperative day one was associated with one-year mortality after burn surgery in elderly patients.
- The postoperative one-year survival rate was lower in elderly burn patients with prognostic nutritional index ≤25.5 than in those with prognostic nutritional index >25.5.
- These results suggest that prognostic nutritional index can provide useful information for the early detection of postoperative mortality in elderly burn patients.

## Background

Burn is one of the most devastating forms of trauma [1]. Elderly patients are particularly susceptible to burn injury because of thinning of the skin, decreased sensation and physical strength, poor vision, mental alteration and coexistence of multiple medical conditions [2–5]. Moreover, burn injury in elderly patients results in poorer outcomes and higher mortality than that in younger patients [6, 7]. During hospital stay, elderly patients who survive a burn injury have higher morbidity, particularly from infectious complications [8]. They are more prone to a prolonged hospital stay and may need post-hospitalization services, such as a skilled nursing facility, nursing home or rehabilitation facility [2–4]. Clinical and therapeutic advancements in burn care, such as implementation of critical care bundles, adequate nutrition and early excision and grafting have markedly increased the lethal dose 50 (LD50) burn size (i.e. burn size associated with a 50% mortality risk) in younger burn patients: the LD50 burn size increased from 49.0% in 1950 to 85.1% in 2010 in patients under 14 years of age [9]. However, the LD50 burn size did not increase markedly in elderly patients: in patients above 65 years of age, it slightly increased from 10.0% in 1950 to 23.1% in 2010 [9]. Therefore, the associated factors for postoperative mortality in elderly burn patients should be evaluated to improve postoperative outcomes.

Prognostic nutritional index (PNI) is based on the patient’s serum albumin level and total lymphocyte count and has been proposed as a method of assessing the perioperative nutritional status, complications and mortality in patients with colorectal cancer [10]. Low PNI is associated with poor outcomes in patients with malignancy [10–14], free flap reconstruction [15], heart failure [16, 17] and kidney transplantation [18]. However, to our knowledge, no study has evaluated the association between perioperative PNI and postoperative mortality in elderly burn patients. In the present study, we aimed to evaluate the independent risk factors, including PNI, for one-year mortality after burn surgery in elderly patients aged ≥65 years.

## Methods

### Patients

In this retrospective study, we reviewed the data of burn patients aged ≥65 years who were admitted to the burn intensive care unit (ICU) for surgery at the Burn Center in Hangang Sacred Heart Hospital, Hallym University, from March 2010 to April 2018. The criteria for admission to the burn ICU were as follows: patients aged 10–64 years with ≥20% total body surface area burned; children aged <10 years and adults aged ≥65 years with ≥10% total body surface area burned; patients with full-thickness burn in ≥10% of the total body surface area; patients with burn involving the eyes, ears, face, hands, feet or perineum that was likely to result in cosmetic or functional impairment; patients with high-voltage electrical burn; and patients with burn complicated by inhalation injury. Patients with incomplete data were excluded. For patients who underwent several burn surgeries, data from the first burn surgery were evaluated. Computerized databases were reviewed to collect the demographic, laboratory and clinical data of the patients. This study was approved by the Institutional Review Board of Hangang Sacred Heart Hospital. The requirement for obtaining written informed consent was waived by the Institutional Review Board. All procedures were carried out in accordance with relevant guidelines and regulations.

### Anaesthetic technique

General anaesthesia was induced with propofol according to our standard institutional protocol [19]. Rocuronium was administered to facilitate tracheal intubation. Anaesthesia was maintained with sevoflurane or desflurane with a mixture of 50% nitrous oxide and 50% oxygen. Mechanical ventilation was performed with a tidal volume of 8–10 ml/kg of the ideal body weight and respiratory rate of 10–14 cycles/min to maintain the end-tidal carbon dioxide tension between 30 and 35 mmHg during burn surgery. Fluid was administered depending on the patient**’**s mean arterial blood pressure, heart rate, blood loss and urine output [19]. In brief, crystalloid was administered at a rate of 6–10 ml/kg/h, and colloid was used when the estimated blood loss was >500 ml during burn surgery. Plasma solution A (CJ Pharmaceutical, Seoul, Korea) or lactated Ringer’s solution was used as crystalloid, and Volulyte (Fresenius Kabi, Bad Homburg, Germany) was used as synthetic colloid during burn surgery. Packed red blood cells (RBCs) were transfused when the haemoglobin concentration was <8 g/dl. The mean arterial blood pressure was maintained at >65 mmHg. If the mean arterial blood pressure was <65 mmHg for at least 5 minutes, additional fluids or vasoactive drugs, such as phenylephrine, ephedrine or norepinephrine, were administered.

### Surgical technique

Surgical planning was usually performed after the burn patient was resuscitated and haemodynamic parameters were restored to acceptable ranges. Surgeries for burn patients included escharotomy, burn wound excision and closure with a cadaveric or split-thickness skin graft. When the burn eschar circumferentially surrounded any body structure (particularly the digits, extremities, abdomen, chest or neck), emergent escharotomy was performed to release the increased interstitial pressure [20]. Depending on the burn depth, the necrotic burn site was excised to a viable depth with tangential excision for smaller burns and fascial excision for larger burns [21]. The standard surgical procedure for rapid and permanent closure of full-thickness burns is split-thickness skin grafting. However, patients with extensive burns often require temporary coverage with a cadaveric skin graft, skin substitutes or a dermal analogue (e.g. Alloderm, Epicel, Biobrane or Integra) because of unavailable or insufficient donor sites [20].

### Data collection

Patient characteristics and preoperative laboratory data that were captured included age, sex, body mass index, comorbidities (diabetes mellitus, hypertension and ischaemic heart disease), Acute Physiology and Chronic Health Evaluation (APACHE) II score, Sequential Organ Failure Assessment (SOFA) score, American Society of Anesthesiologists (ASA) physical status, total body surface area burned, burn type (flame, scalding, contact and other burns), presence of inhalation injury, preoperative laboratory data (haemoglobin level, platelet count, lymphocyte count, serum albumin level, PNI and serum creatinine level) and interval between admission and the first operation. Inhalation injury was diagnosed clinically based on the circumstances in which the burn was obtained, including a history of burn in an enclosed space, physical findings (facial burn, singed facial hairs or carbonaceous sputum), elevated carboxyhaemoglobin or bronchoscopic findings (airway oedema, mucosal necrosis, ulceration of bronchi or soot in the airway). Intraoperative variables included anaesthesia duration, surgery duration, crystalloid amount, colloid amount and RBC transfusion rate and amount. Postoperative laboratory data included the lymphocyte count, serum albumin level and PNI, which were assessed on postoperative day one.

### Definition of PNI on postoperative day one

PNI on postoperative day one was defined as the value calculated on postoperative day one using the following equation: 10 × serum albumin level (g/dL) + 0.005 × total lymphocyte count (per mm^3^) [22].

### Primary and secondary outcomes

The primary outcome was the identification of risk factors, including PNI on postoperative day one, for one-year mortality after burn surgery in elderly patients. The secondary outcome was the comparison of one-year mortality between the two groups, which were dichotomized according to the optimal cut-off PNI value on postoperative day one for predicting postoperative one-year mortality in elderly burn patients*.*

### Statistical analysis

Continuous variables are expressed as mean ± standard deviation. They were compared between the two groups using Student’s *t*-test or Mann–Whitney *U* test, as appropriate. Categorical variables are expressed as number (percentage). They were compared between the two groups using the chi-square test or Fisher***’***s exact test, as appropriate. The Cox proportional hazard model was used for univariate and multivariate analyses of the risk factors associated with one-year mortality after burn surgery in elderly patients. The identified factors with a *p* value <0.05 in the univariate Cox logistic regression analysis were included in the multivariate Cox logistic regression analysis. The receiver operating characteristic curve analysis was performed to evaluate the predictive ability of PNI on postoperative day one for postoperative one-year mortality in elderly burn patients. The value with the highest sensitivity and specificity was set as the optimal cut-off PNI value. The Kaplan–Meier survival analysis, with a log-rank test, was performed to compare the postoperative one-year survival rates between the two groups, which were dichotomized according to the optimal cut-off PNI value on postoperative day one for predicting postoperative one-year mortality. A *p* value <0.05 was considered significant. All statistical analyses were performed using SPSS for Windows (version 22.0; IBM-SPSS Inc., Armonk, NY, USA).

## Results

**Figure 1.**
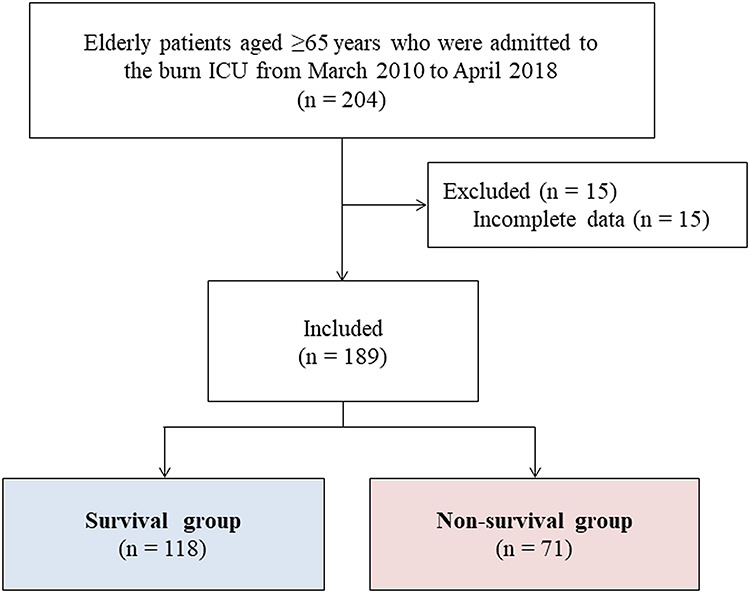
Flow diagram of the study participants. *ICU* intensive care unit

A total of 204 patients were considered for the study. Fifteen patients with incomplete data were excluded. The reasons for exclusion were a lack of preoperative or postoperative PNI data for six and nine patients, respectively. In total, 189 patients were included in the study (Figure 1). The overall mortality rate after burn surgery in elderly patients aged ≥65 years who were admitted to the burn ICU was 37.6% (71 of 189 patients).

Table 1 shows the patient characteristics and preoperative laboratory data, in which the sex, APACHE II score, SOFA score, ASA physical status, total body surface area burned, burn type, inhalation injury, platelet count, serum albumin level, PNI, serum creatinine level and interval between the admission and the first operation were significantly different between the survival and non-survival groups. For intraoperative variables, crystalloid and RBC transfusion amounts were significantly different between the two groups (Table 2). In addition, the lymphocyte count, serum albumin level and PNI on postoperative day one were significantly different between the two groups (Table 2).

**Table 1:** Patient characteristics and preoperative laboratory data

**Variables**	**Survival group (n = 118)**	**Non-survival group (n = 71)**	***P* value**
Age, years	74.7 ± 7.2	76.0 ± 6.0	0.207
Male sex	67 (56.8)	58 (81.7)	<0.001
Body mass index, kg/m^2^	22.9 ± 3.2	23.2 ± 4.0	0.548
Diabetes mellitus	20 (16.9)	19 (26.4)	0.106
Hypertension	57 (48.3)	34 (47.8)	0.956
Ischaemic heart disease	13 (11.0)	10 (14.1)	0.532
APACHE II score	9.0 (7.0–11.0)	12.0 (10.0–17.0)	<0.001
SOFA score	1.0 (1.0–2.0)	3.0 (1.0–5.0)	<0.001
ASA physical status			<0.001
≤2	68 (57.6)	21 (29.6)	
≥3	50 (42.4)	50 (70.4)	
Total body surface area burned, %	20.0 (10.0–29.5)	35.0 (20.0–50.0)	<0.001
Burn type			0.004
Flame burn	70 (59.3)	56 (78.9)	
Scalding burn	30 (25.4)	6 (8.5)	
Contact burn	18 (15.3)	7 (9.9)	
Other burns^a^	0 (0)	2 (2.8)	
Inhalation injury	20 (16.9)	21 (29.6)	0.041
Preoperative laboratory data			
Haemoglobin, g/dl	11.0 ± 2.1	11.2 ± 2.6	0.675
Platelet count, 10^9^/L	223.0 (149.8–350.3)	139.0 (94.0–211.0)	<0.001
Lymphocyte count, per mm^3^	1024.7 ± 519.6	891.0 ± 556.7	0.115
Albumin, g/dl	2.7 (2.4–2.9)	2.3 (2.0–2.6)	<0.001
PNI	30.7 (28.8–35.4)	27.4 (23.5–31.0)	<0.001
Creatinine, mg/dl	0.7 (0.5–0.9)	0.9 (0.7–1.3)	<0.001
Interval between admission and first operation, days	6.8 ± 7.2	4.1 ± 4.6	0.002

Data are shown as number (%), mean ± standard deviation or median (interquartile range), as appropriate. *APACHE* Acute Physiology and Chronic Health Evaluation, *SOFA* Sequential Organ Failure Assessment, *ASA* American Society of Anesthesiologists, *PNI* prognostic nutritional index

^a^Other burns included spark, chemical and steam burns

**Table 2:** Intraoperative variables and postoperative laboratory data

**Variables**	**Survival group (n = 118)**	**Non-survival group (n = 71)**	***P* value**
Anaesthesia duration, minutes	129 ± 52	124 ± 38	0.548
Operation duration, minutes	84 ± 50	87 ± 36	0.669
Crystalloid amount, ml/kg	16.4 (10.7–24.8)	21.0 (13.7–29.5)	0.028
Colloid amount, ml/kg	9.6 ± 5.6	10.6 ± 7.1	0.265
RBC transfusion rate	105 (89.0)	68 (95.7)	0.175
RBC transfusion amount, unit	3.0 (2.0–5.0)	4.0 (3.0–6.0)	<0.001
Laboratory data on postoperative day one			
Lymphocyte count, per mm^3^	795 (540–1123)	530 (400–890)	<0.001
Albumin, g/dl	2.6 (2.4–2.8)	2.3 (2.0–2.5)	<0.001
PNI	29.9 (26.5–33.7)	25.5 (23.0–28.4)	<0.001

Data are shown as number (%), mean ± standard deviation or median (interquartile range), as appropriate. *RBC* red blood cell, *PNI* prognostic nutritional index

The univariate Cox logistic regression analysis revealed that the PNI on postoperative day one, sex, APACHE II score, SOFA score, ASA physical status, total body surface area burned, scalding burn, inhalation injury, preoperative serum creatinine level, interval between the admission and the first operation, crystalloid amount and RBC transfusion amount were significantly associated with postoperative one-year mortality. The multivariate Cox logistic regression analysis revealed that PNI on postoperative day one (hazard ratio (HR) = 0.872; 95% CI = 0.812–0.936; *p* < 0.001), SOFA score (HR = 1.112; 95% CI = 1.005–1.230; P = 0.04), ASA physical status (HR = 2.064; 95% CI = 1.211–3.517; *p* = 0.008), total body surface area burned (HR = 1.017; 95% CI = 1.003–1.032; *p* = 0.015) and preoperative serum creatinine level (HR = 1.386; 95% CI = 1.058–1.816; *p* = 0.018) were significantly associated with one-year mortality after burn surgery in elderly patients (Table 3).

**Table 3:** Univariate and multivariate Cox regression analyses for risk factors associated with one-year mortality after burn surgery in elderly patients

**Variables**	**Univariate analysis**		**Multivariate analysis**	
	HR (95% CI)	*P* value	HR (95% CI)	*P* value
Age	1.017 (0.986–1.050)	0.288		
Male sex	2.735 (1.498–4.995)	0.001		
Body mass index	1.014 (0.949–1.083)	0.686		
Diabetes mellitus	1.629 (0.963–2.755)	0.069		
Hypertension	0.977 (0.613–1.557)	0.977		
Ischaemic heart disease	1.171 (0.600–2.286)	0.643		
APACHE II score	1.107 (1.068–1.148)	<0.001		
SOFA score	1.265 (1.163–1.377)	<0.001	1.112 (1.005–1.230)	0.040
ASA physical status				
≤2	1.0		1.0	
≥3	2.739 (1.644–4.564)	<0.001	2.064 (1.211–3.517)	0.008
Total body surface area burned	1.043 (1.030–1.056)	<0.001	1.017 (1.003–1.032)	0.015
Burn type				
Flame burn	1.0			
Scalding burn	0.302 (0.130–0.700)	0.005		
Contact burn	0.530 (0.242–1.164)	0.114		
Other burns^a^	3.866 (0.927–16.132)	0.064		
Inhalation injury	1.791 (1.076–2.983)	0.025		
Preoperative haemoglobin	1.023 (0.924–1.134)	0.657		
Preoperative creatinine	1.792 (1.452–2.211)	<0.001	1.386 (1.058–1.816)	0.018
Interval between admission and first operation	0.928 (0.879–0.981)	0.008		
Anaesthesia duration	0.999 (0.994–1.004)	0.643		
Crystalloid amount	1.025 (1.004–1.046)	0.020		
RBC transfusion amount	1.246 (1.139–1.363)	<0.001		
PNI on postoperative day one	0.805 (0.759–0.854)	<0.001	0.872 (0.812–0.936)	<0.001

The multivariate logistic regression analysis was performed for all factors (i.e. sex, *APACHE II score*, SOFA score, ASA physical status, total body surface area burned, inhalation injury, preoperative creatinine, interval between the admission and the first operation, crystalloid amount, RBC transfusion amount and PNI on postoperative day one) with *p* < 0.05 in univariate logistic regression analyses. *HR* hazard ratio, *APACHE* Acute Physiology and Chronic Health Evaluation, *SOFA* Sequential Organ Failure Assessment, *ASA* American Society of Anesthesiologists, *RBC* red blood cell, *PNI* prognostic nutritional index

^a^Other burns included spark, chemical and steam burns

**Figure 2.**
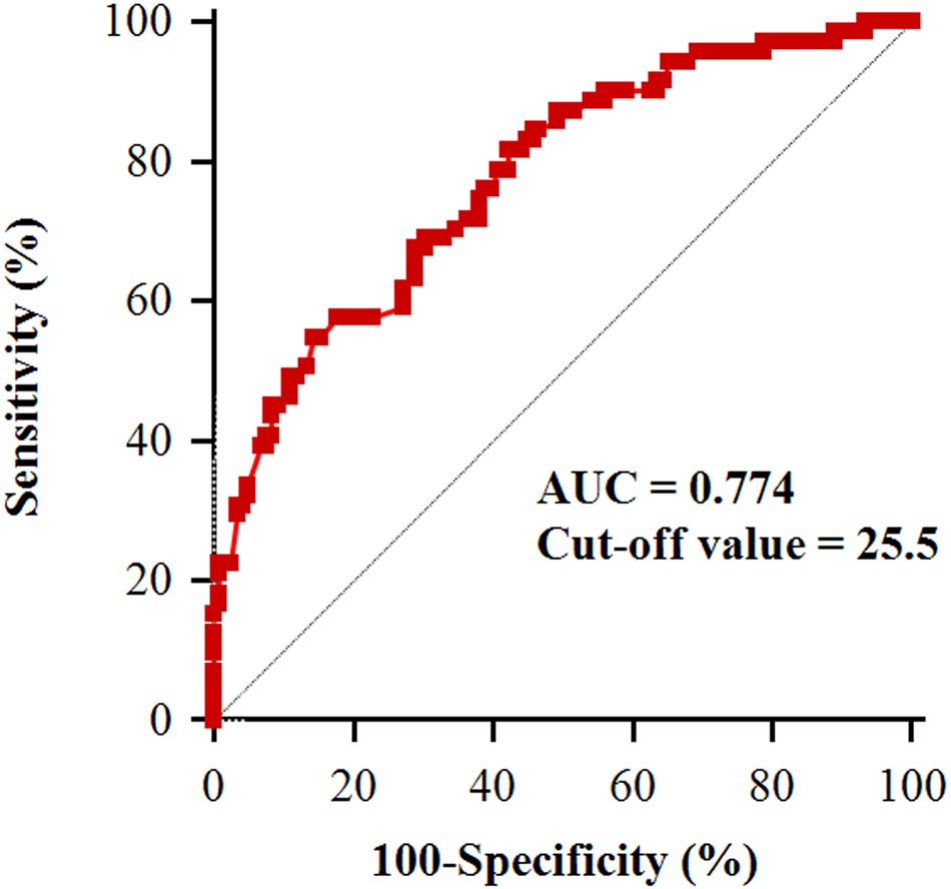
Receiver operating characteristic curve analysis of the prognostic nutritional index on postoperative day one to predict one-year mortality after burn surgery in elderly patients aged ≥65 years. The area under the curve is 0.774, with an optimal cut-off value of 25.5. *AUC* area under the curve

**Figure 3.**
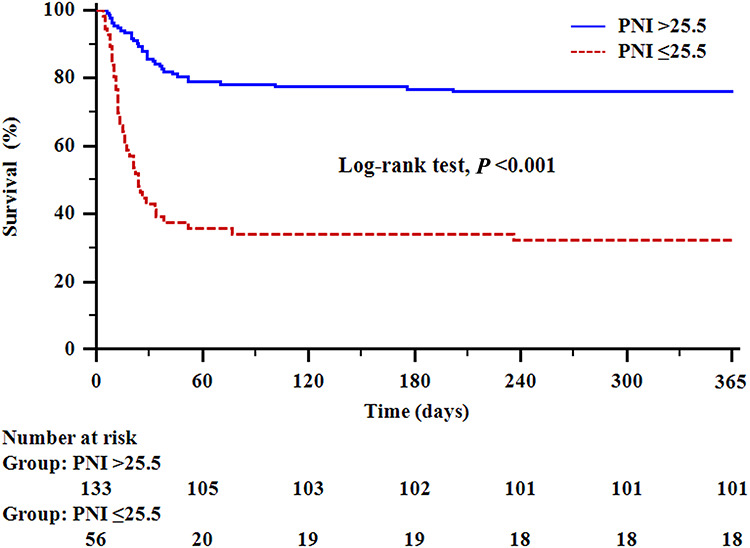
Kaplan–Meier curve of one-year mortality according to the optimal cut-off prognostic nutritional index (PNI) value on postoperative day one. The solid blue line indicates one-year survival in elderly burn patients with PNI >25.5. The red dashed line indicates one-year survival in elderly burn patients with PNI ≤25.5. The one-year survival rate was significantly lower in elderly burn patients with PNI ≤25.5 than in those with PNI >25.5

Figure 2 shows the predictive value of PNI on postoperative day one for postoperative one-year mortality in elderly patients. The area under the curve for PNI on postoperative day one was 0.774 (95% CI = 0.708–0.832), with a sensitivity of 54.9% and specificity of 85.6%. The optimal cut-off PNI value on postoperative day one in predicting postoperative one-year mortality was 25.5. Figure 3 shows the Kaplan–Meier survival curve in elderly burn patients with PNI >25.5 and ≤25.5. The one-year survival rate was significant lower in elderly patients with PNI ≤25.5 than in those with PNI >25.5 (32.1% *vs* 75.9%, *p* < 0.001).

## Discussion

In this study, the incidence of one-year mortality after burn surgery in elderly patients who were admitted to the burn ICU was 37.6%. Lower PNI on postoperative day one, higher SOFA score, higher ASA physical status score, greater total body surface area burned and higher preoperative serum creatinine level were associated with one-year mortality after burn surgery in elderly patients. The optimal cut-off PNI value on postoperative day one for predicting postoperative one-year mortality was 25.5. Furthermore, the one-year survival rate was significantly lower in elderly burn patients with PNI ≤25.5 than in those with PNI >25.5.

Burns can cause major multi-systemic stress because of burn injury-related fluid shift and subsequent fluid resuscitation, systemic inflammatory response and high metabolic rate [23]. Importantly, elderly patients may have limited physiological reserves and multiple significant comorbidities [1]. Consequently, burn treatment outcomes are poor in elderly patients compared to younger patients [4, 24–26]. However, burn management of elderly patients remains a challenge from clinical and rehabilitative perspectives. Therefore, perioperative risk evaluation is important to reduce poor postoperative outcomes in elderly burn patients. To the best of our knowledge, this is the first study to assess the risk factors, including PNI, for one-year mortality after burn surgery in elderly patients.

We found that lower PNI on postoperative day one was an independent risk factor for postoperative one-year mortality in elderly burn patients. The exact mechanisms by which the PNI is related to the postoperative prognosis remain incompletely understood. Several potential mechanisms have been reported. First, serum albumin level is widely used to evaluate the nutritional and systemic inflammatory aspects of patients [27–30], and it correlates with postoperative complications, including burns [31–33]. A postoperative albumin decline can reflect the magnitude of surgical trauma and is related to adverse clinical outcomes, such as postoperative complications and prolonged hospital stay [34]. A decrease in the albumin level is driven by a potential systemic inflammatory response [29]. Ishida *et al.* suggested that the serum albumin level is correlated with systemic inflammation in burn patients [30]. Aguayo-Becerra *et al.* reported that hypoalbuminemia was strongly associated with greater burn severity and higher mortality [31]. Second, because lymphocytes play a pivotal role in immune response, their functions and numbers are profoundly altered after the occurrence of sepsis and other acute injuries, such as severe trauma, extensive burns or major surgeries [35]. Lymphopenia has been reported in thermally injured patients [36]. Failure to re-establish a normal lymphocyte count after traumatic injury and sepsis has been associated with increased mortality [37–39]. Osuka *et al.* also reported that early decrease in the lymphocyte count was a poor prognostic factor in burn patients [40]. Based on these considerations, we hypothesized that PNI would be a prognostic factor for mortality in burn patients. We considered that PNI on postoperative day one could more effectively reflect postoperative surgical stress, including blood loss, catabolic inflammatory status and decreased hepatic protein synthesis, compared to preoperative PNI, particularly in burn patients [41]. In the present study, PNI, which is an immune indicator as well as a nutritional and inflammatory indicator, was assessed on postoperative day one and found to be associated with one-year mortality after burn surgery in elderly patients*.*

We found that the optimal cut-off PNI value on postoperative day one for predicting one-year mortality in elderly burn patients was 25.5. However, the cut-off PNI value in our study was much lower than in other studies. The cut-off PNI values for overall survival in stomach cancer patients who required gastrectomy were 49.7, 44.7 and 48 in studies by Nozoe *et al.*, Watanabe *et al.* and Migita *et al.*, respectively [42–44]. Tominaga *et al.* reported that the overall survival, five-year relapse-free survival and cancer-specific survival rates were significantly worse in elderly colon cancer patients with PNI <42.4 [45]. The cut-off PNI values in gastrointestinal cancer patients ranged from 40 to 49.7 [46]. These discrepancies in the cut-off values between patients with burn and those with other diseases may, at least in part, be due to the specific characteristics of burns. Thermal injury leads to tissue destruction with capillary leak, oedema formation and profound hypovolemia. Initial fluid resuscitation with a large volume of crystalloid could cause haemodilution and hypoalbuminaemia, which frequently occur after a severe burn injury [23]. Therefore, the cut-off PNI value for predicting postoperative one-year mortality may be relatively low in our study population*.*

We found that a higher SOFA score was associated with one-year mortality after burn surgery in elderly patients. Consistent with our results, a previous study showed that the SOFA score was associated with inpatient mortality in severe burn injury [47]. Moreover, the SOFA score is useful to assess organ dysfunction in burn injury patients [48]. Based on these considerations, burn-induced organ dysfunction could be associated with postoperative mortality in elderly patients. Therefore, preoperative evaluation of the SOFA score could be used to predict one-year mortality after burn surgery in elderly patients.

In this study, an ASA physical status score ≥3 was associated with one-year mortality after a burn injury in elderly patients. The ASA physical status classification is a method of characterizing a patient’s operative risk on a scale of 1–6, where 1 is normal health and 5 is moribund (6 = need for organ transplant). An ASA physical status of 3 indicates “a patient with severe system disease”. The mortality rate increased with an increase in the ASA physical status score. Approximately 0.02% of patients with an ASA physical status score of 1, 0.14% with a score of 2, 1.41% with a score of 3, 11.14% with a score of 4 and 50.87% with a score of 5 died 30 days postoperatively [49]. Therefore, we believe that meticulous perioperative burn management is necessary to reduce the risk of poor postoperative outcomes in elderly patients with an ASA physical status score ≥3.

In the present study, the total body surface area burned was associated with one-year mortality after burn surgery. The average total body surface area burned was different between the survivor and non-survivor groups (20.0% vs. 35.0%) in our population. Similarly, other studies demonstrated that the mortality rate increased in patients with burns to a higher percentage of the total body surface area [5, 50–52]. Burns to a higher percentage of the total body surface area often lead to persistent catabolic and immunocompromised states, which can increase the risks of wound infection, sepsis and multiple organ failure [53]. Furthermore, the treatment of such patients remains a challenge because of the lack of autologous skin and need for more extensive and multiple operations [54]. As the total body surface area burned increases, the rate of mortality in elderly burn patients tends to increase.

Elevated preoperative serum creatinine level increased the risk of one-year mortality in elderly burn patients. Preoperative serum creatinine level may increase because of renal damage and decreased blood flow, particularly in patients with inappropriate initial fluid resuscitation. Yang *et al.* reported that increased serum creatinine level at admission was associated with acute kidney injury development in patients with major burn injury [55]. Acute kidney injury is a major contributor to morbidity and mortality in patients with burns [56]. Because elevated preoperative serum creatinine and subsequent renal damage are associated with mortality after burn surgery, preoperative evaluation of serum creatinine is considered to play an important role in evaluating the postoperative outcomes of patients who have had burn surgery.

Our study had several limitations. First, it was retrospective in nature. Although the inevitable selection bias was reduced by considering all risk factors potentially influencing mortality after burn surgery, the possibility of this bias in our retrospective analysis cannot be excluded. Particularly, measurement of the frailty score in elderly patients may be useful in evaluating postoperative outcomes of burn surgery. However, we could not evaluate the frailty score because of the incomplete data on the psychological, social and functional conditions of the patients included in this retrospective study. Second, our burn patient cohort was recruited from a single experienced centre, and our results need to be interpreted accordingly.

## Data Availability

Data are available from the authors upon reasonable request.

## Conclusions

PNI on postoperative day one was associated with one-year mortality after burn surgery in elderly patients. The postoperative one-year survival rate was lower in elderly burn patients with PNI ≤25.5 than in those with PNI >25.5. These results suggest that PNI can provide useful information for the early detection of postoperative mortality in elderly burn patients.
